# Folding and Unfolding in the Blue Copper Protein Rusticyanin: Role of the Oxidation State

**DOI:** 10.1155/2007/54232

**Published:** 2007-08-29

**Authors:** Luis A. Alcaraz, Javier Gómez, Pablo Ramírez, Juan J. Calvente, Rafael Andreu, Antonio Donaire

**Affiliations:** ^1^Instituto de Biología Molecular y Celular, Universidad Miguel Hernández de Elche, Edificio Torregaitán, Avda. de la Universidad s/n, 03202 Elche, Alicante, Spain; ^2^Departamento de Química Física, Universidad de Sevilla, C/Professor García González s/n, 41012 Sevilla, Spain; ^3^Departamento de Química Inorgánica, Facultad de Química, Universidad de Murcia, Campus Universitario, Apdo. 4021, 30071 Murcia, Spain

## Abstract

The unfolding process of the blue copper protein rusticyanin has been studied from the structural and the thermodynamic points of view at two pH values (pH 2.5 and 7.0). When Rc unfolds, copper ion remains bound to the polypeptide chain. Nuclear magnetic resonance data suggest that three of the copper ligands in the folded state are bound to the metal ion in the unfolded form, while the other native ligand is detached. These structural changes are reflected in the redox potentials of the protein in both folded and unfolded forms. The affinities of the copper ion in both redox states have been also determined at the two specified pH values. The results indicate that the presence of two histidine ligands in the folded protein can compensate the change in the net charge that the copper ion receives from their ligands, while, in the unfolded protein, charges of aminoacids are completely transferred to the copper ion, altering decisively the relative stability of its two-redox states.

## 1. INTRODUCTION

Copper is essential for life. It participates in many biological events [1–3]. After iron, nature has chosen this metal ion as the second element in participating in electron transfer chains [4–6]. Its thermodynamics and the kinetic easiness to oxidize/reduce itself allow copper containing proteins to play a crucial role as electron carriers in many vital processes. Copper proteins possess well-designed active centers that finely tune metal ion redox properties [7, 8]. In turn, ligands, geometries, and surroundings of the copper ion are a direct consequence of protein scaffold. Therefore, protein folding stabilizes one copper oxidation state versus the other one, resulting in a metal ion biologically active. Protein redox potentials are a consequence of the different affinities of the polypeptide chain for the metal ion in both redox states [9, 10]. It follows the relevance of studying the formation of the functional (folded) holoprotein, in both the reduced and the oxidized states, from the structural and functional points of view. Taking into account that the holoprotein can be formed as an unfolded protein prior to copper uptake inside the cell [11], it also follows the relevance of the same kind of studies with the unfolded protein. This last point is still a subject of debate [10, 12].

**Figure 1:**
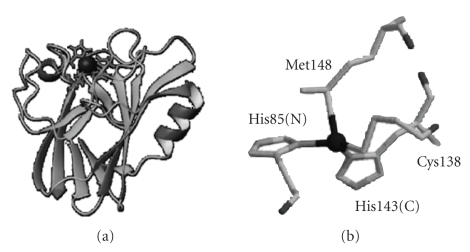
(a) Ribbon backbone of rusticyanin. The arrangement of the *β*-strands forming the *β*-barrel is common to all BCPs. (Helixes shown in the figure are exclusive of Rc.) (b) Active site of Rc (common to most of BCPs). Ligand numeration corresponds to Rc aminoacid sequence. HisN (His85 in Rc) is far away in the primary sequence from the other three coordinating residues (Cys, His143(C), and Met).

Blue copper proteins (BCPs) are relatively small soluble copper proteins [7, 14, 15]. Their characteristic colour arises from a charge transfer ligand-to-metal band between a cysteinyl sulphur atom and the copper(II) ion [8, 16]. All BCPs present the so-called Greek key *β*-barrel as a common feature (Figure 1(a)). This is a rigid structure essentially kept by an extended network of hydrogen bonds and tertiary interactions between amino acid side chains. This rigidity is transmitted to the metal ion and is essential for the electron transfer. The copper ion is bound to a cysteine and two histidine residues. A fourth ligand completes the copper coordination sphere for most BCPs [17]. This fourth ligand is usually a methionine (Figure 1(b)). In all BCPs, the ligands Cys, Met, and one of the histidines (hereafter called HisC, due to its proximity to the C-terminal end) are close among them in the primary sequence, while the other histidine, HisN, is far away from them in the amino acid chain (Figure 1(b)).

Table 1 shows the redox potentials of some representative BCPs [13]. They are all encompassed between 120 and 620 mV, while that of the pair Cu(II)/Cu(I) in aqueous solution is 150 mV, substantially lower than most of them. Thus, these proteins considerably increase the stability of the reduced versus the oxidized state of the copper ion. Rusticyanin (Rc) is, by far, the BCP with the highest capability in this respect. Rc is the most abundant soluble protein of the Gram-negative bacterium *Acidithiobacillus ferrooxidans* (*Af*) [18–20]. This organism lives in media that are very acidic and containing high concentrations of metals. Consequently, Rc is extraordinarily stable at low pH values.

According to the well-established “rack state” [1] or “entatic” [21] mechanism, protein folding in BCPs imposes a coordination geometry atypical to the copper(II) ion. Oxidized copper would then be destabilized, increasing the redox potential of the metal ion. Nevertheless, the BCP azurin presents a redox potential in the unfolded state higher than that of the folded protein (450 *versus* 276 mV) [10, 22]. So, apparently, the unfolded protein stabilizes the reduced copper in a more efficient way, and protein folding seems not to be so crucial in keeping the redox potential of this BCP.

Here we present an exhaustive study of the unfolding process of rusticyanin by means of guanidinium chloride (GdmCl). We have previously characterized the structural facets of the Rc unfolding process [12, 23, 24]. We expose now the parallel changes produced in the Rc redox potential (i.e., the different affinity of the metal ion in the two redox states). Rc retains copper(I) and copper(II) in the unfolded form. Moreover, the redox potential of this pair for the unfolded Rc is slightly higher at low pH values than the analogous pair for the folded form. We demonstrate that the folding scaffold not only rises the protein affinity for the metal ion in whatever oxidation state, but also, and mainly, hampers drastic changes in its redox properties when pH is modified.

## 2. EXPERIMENTAL

### 2.1. Protein preparation

Recombinant rusticyanin was obtained from BL21(DE3) *Escherichia coli* containing the Rc plasmid [19]. Bacteria cultures were grown in suitably modified M9 medium [25]. Samples for all techniques were prepared as previously described [25]. Conditions for all experiments (unless otherwise indicated) were formiate (pH 2.5) or phosphate (pH 7.0) buffers 10 mM, 298 K.

### 2.2. Uv-visible and nuclear magnetic resonance spectroscopies

Uv-visible data were collected in a UV-visible spectrophotometer (Shimadzu UV-1603). Copper titrations were carried out starting from the apoprotein (2–5 ×10^–5^ M) and adding the corresponding aliquots of copper solutions up to protein saturation. Buffers used were formic acid 10 mM and Tris chloride 10 mM at pH 2.5 and 7.0, respectively. For the folded protein, copper(II) titrations were performed by following the absorption band at 592 nm (*ε* = 2240 M^–1^ cm^–1^) [26]. For the unfolded protein, the formation of the complex was followed by the absorbance at 270 nm (*ε* = 2000 M^–1^ cm^–1^) due to the copper(II)-S *γ* Cys-band charge transfer band, as we have previously described [12]. This absorbance is added to the intrinsic absorption of rusticyanin at 280 nm.

**Scheme 1:**
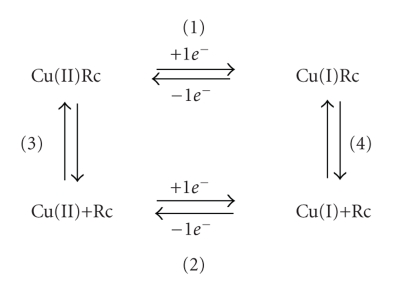
Thermodynamic cycle associated to the redox potential of CuRc. Redox potentials (equations 1 and 2) are given versus the normal pair H^+^, H_2_ in standard conditions. Copper(II) redox potential (equation 2) is 150 mV (Δ*G* ^0^= −3.45 kcal/mol). All values were determined at 298 K.

The affinity constants of rusticyanin for the reduced species were deduced applying the thermodynamic cycle shown in Scheme 1 (see Section 3). Throughout all the text, values of equilibria 3 and 4 are always referred to the formation of the corresponding holoforms (affinity constants) and, consequently, they are given in units of M^−1^. All experiments were performed at 298 K.

In nuclear magnetic resonance (NMR) measurements, eight percent of D_2_O was added to the samples used (phosphate buffer 10 mM, pH 5.5, 298 K). All the experiments were performed as described in [25].

### 2.3. Cyclic voltammetry

Voltammetric experiments were carried out with an Autolab PGSTAT30 (Echo Chemie) in a low volume (∼3mL) electrochemical cell with a three-electrode configuration [27]. The working electrode was either a gold disk modified with 4-mercaptopyridine (measurements at pH 7.0), or a carbon paste surface modified with 4, 4′-bipyridyl (measurements at pH 2.5). Electrolyte solutions contained 0.1 M sodium phosphate buffer, 0.1 mM rustcyanin, and, in the studies of the unfolded protein, 8 M guanidinium chloride. All measurements were carried out at 25 ± 0.2°C. Given electrode potential values are referred to the normal hydrogen electrode (NHE) scale. Signals were reversible both for folded and unfolded forms. The estimated error of potential measurement was lower than ± 15 mV in all cases (the errors of each measurement are specifically given in Section 3).

## 3. RESULTS AND DISCUSSION

### 3.1. The unfolding process: structural and dynamic characterization

**Figure 2:**
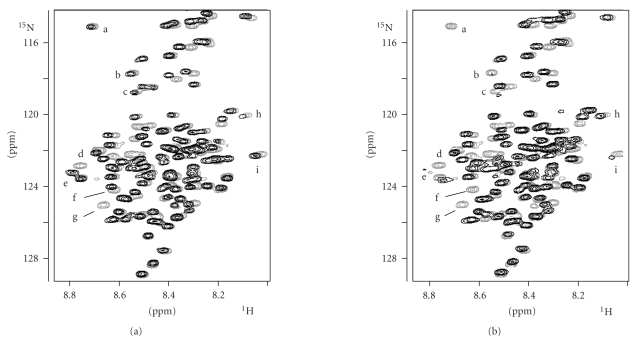
^1^H−^15^N HSQC spectrum of unfolded rusticyanin: (a) ApoRc (black) versus Cu(I)Rc (light grey); (b) Cu(II)Rc (black) versus Cu(I)Rc (light grey). Guanidinium chloride concentration was 6.0 M, pH 5.5, 298 K. Signal*a* − *i* change their position and/or disappear from one species to the other, that is, they are close to the copper ion.

We have previously characterized the chemical unfolding process of Rc by different biophysical techniques [12, 23, 24]. NMR permits to obtain dynamical and structural information not provided by other spectroscopies. In fact, diffraction crystallography cannot be applied to partially or totally protein unfolded states and then NMR becomes the most powerful structural technique. Figure 2 shows the ^1^H −^15^N HSQC spectra obtained from three solutions containing apoRc, Cu(I)Rc, and Cu(II)Rc after adding GdmCl until complete protein unfolding. The three spectra are nearly superimposable, indicating that, essentially, the same species are present in the three samples. When comparing apoRc and Cu(I)Rc (Figure 2(a)), a set of few signals (labelled as *a* − *i*) modify their chemical shifts. These small local changes indicate that copper(I) remains bound to the unfolded rusticyanin. In the ^1^H−^15^N HSQC spectrum of copper(II) rusticyanin (black line, Figure 2(b)), the intensities of the same signals (*a*−*i*) are markedly diminished, or they are even lost, due to the paramagnetism of copper(II) [28]. Thus, the metal ion is bound to the unfolded polypeptide chain at the same residues in whatever oxidation state. Unfortunately, at this stage, the low resolution of the spectra did not allow us to unequivocally identify the specific residues bound to the copper ion. The redox activity of the unfolded rusticyanin (see below) also demonstrates that copper is bound to this form of the protein.

**Figure 3:**
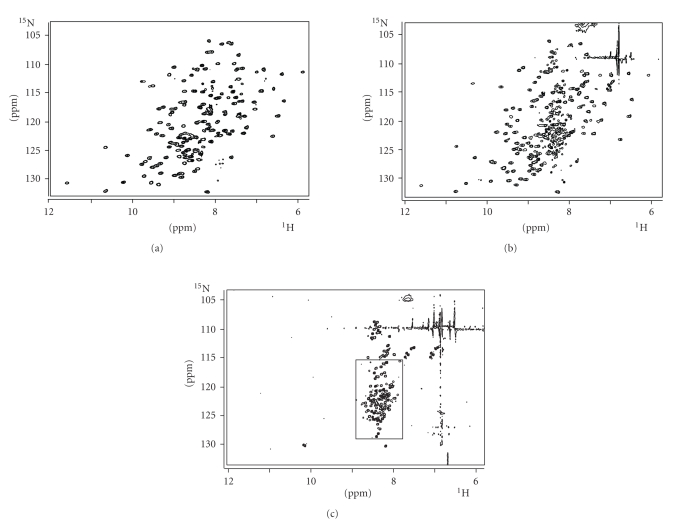
^1^H^ — 15^N HSQC spectra of Cu(I)Rc at three different concentrations of GdmCl: (a) 0 M (folded protein); (b) 2.1 M; and (c) 6.0 M (completely unfolded protein). The rest of the conditions are the same as in Figure 2. The square in (c) corresponds to the expansion shown in light grey in Figure 2(b).

**Figure 4:**
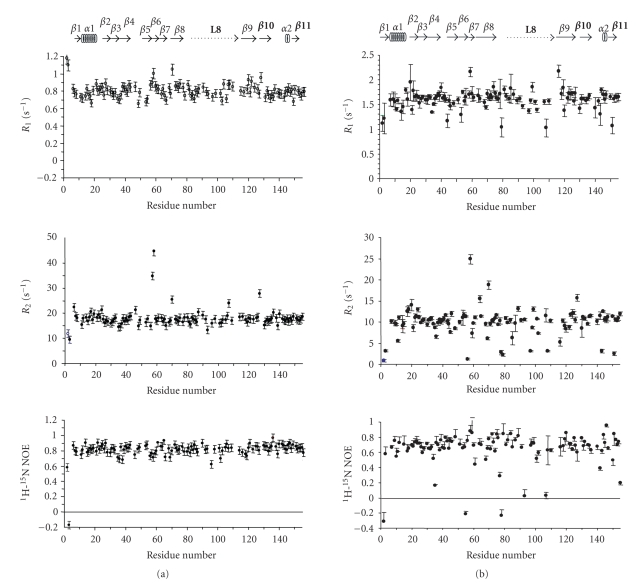
Relaxation parameters (*R*_1_, *R*_2_, and ^1^H−^15^N NOE values) for (a) the completely folded Cu(I)Rc [25] and (b) Cu(I)Rc partially unfolded at 2.1 M GdmCl. At the top of the figure, the secondary structural elements of Rc (*β* -strands: *β*1 − *β*11, *α* -helixes:*α* 1 and *α* 2, and the long loop L8) are indicated. The elements cited in the text are in bold.

Figure 3 shows the ^1^H−^15^N HSQC of Cu(I)Rc at different guanidinium chloride concentrations. In absence of the denaturant agent, only the folded species (characterized by a large dispersion of the signals) is present (Figure 3(a)). On the contrary, only the unfolded species are observed at GdmCl 6M (Figure 3(c)). At GdmCl 2.1M (Figure 3(b)), both species are present in different ratios (*ca. * 3 : 2 for the folded versus the unfolded species, resp.). At this step, the folded species essentially has the same chemical shifts as the completely folded species, indicating that the tridimensional protein structure is kept. We have determined the mobility features of this species. Figure 4 displays the comparison of the relaxation data (*R*_1_, *R*_2_, and ^1^H−^15^N NOE) for the completely folded Rc and the species present at a GdmCl 2.1M concentration. As observed, folded Rc at these conditions is much more mobile than the completely folded species (i.e., in absence of GdmCl). In other words, GdmCl induces the opening of Rc tridimensional structure.

Two main conclusions are immediately deduced from the relaxation data. First, with the exception of the N -terminal domain, the largest mobile region of Cu(I)Rc at GdmCl 2.1 M is that encompassing residues 80–120 (Figure 4(b)). These residues correspond to a long loop present not only in Rc, but also in all BCPs. It is interesting to remark that the fist copper ligand in the primary sequence, HisN (His85 in Rc, Figure 1(a)), belongs to this loop [29]. The dynamic properties of this ligand itself indicate that this histidine undergoes exchange processes. Deuterium exchange experiments also indicate that amide protons of this domain are highly exposed to the solvent in these conditions (data not shown), corroborating that this loop is prone to open. By resuming, HisN is more labile when the protein starts to unfold.

On the contrary, the *C* -terminal domain (amino acids 120–155, encompassing the last two *β *-strands) is one of the most rigid domains of the protein even when GdmCl is added (Figure 4(b)). Moreover, most of the amide protons in these regions essentially remain unchanged upon addition of GdmCl (data not shown). Both data indicate that the extreme *C* -terminal is one of the most protected domains of Rc, even in the presence of the denaturant agent. The three last ligands of the metal ion (Cys138, His143, and Met148, see Figure 1(b)) belong to this domain [29].

In summary, when the denaturant agent is added, the interaction of the copper ion with HisN starts to weaken, while that kept with the other three ligands is not essentially altered. This strongly suggests that these three ligands remain bound to the metal ion when the protein unfolds, while HisN is lost from the copper coordination.

**Figure 5:**
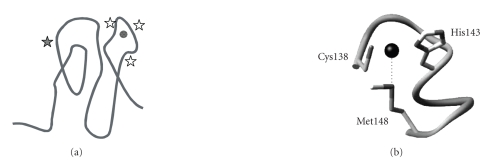
(a) Schematic view of the unfolded Rc. White stars show the position of Cys138, His143(C), and Met 148 (metal ligands). The shaded star indicates the position of His85(N) (not coordinated to the metal ion in the unfolded state). (b) Schematic view of the active site in unfolded Rc. Ligands that remain coordintated to the copper ion are indicated (white stars in (a)).

This conclusion is totally supported by comparison between the present results and previous data obtained from the BCP azurin. In fact, the unfolding process of azurin has been amply studied [10, 30, 31]. It has been demonstrated that HisN in azurin is detached from the copper ion when protein unfolds, while the other three ligands remain bound to it. Moreover, the redox potential of the unfolded rusticyanin (450 mV, see below) and azurin (456 mV, [22]) is the same within the experimental error, indicating that copper possesses a similar coordination .sphere in both unfolded proteins. We can conclude that the same process occurs for these two similar BCPs. When Rc (or azurin) unfolds, copper ion remains bound to the three ligands close in the primary sequence (Cys, HisC, and Met), two of which belong to defined secondary structure elements of the protein (the last two *β* -strands of the *β* -barrel, see Figure 4). On the contrary, the first ligand (HisN), far away in the primary sequence and located in a flexible loop of the protein, detaches from copper upon unfolding. Figure 5 displays a schematic view of copper coordination in unfolded Rc.

### 3.2. Functional aspects: redox potentials, and relative copper-protein affinities

Values of redox potentials of a holoprotein reflect the different affinity of the apoprotein for the two redox states of a metal ion. The corresponding thermodynamic cycle applied to CuRc is shown in Scheme 1. We have studied the equilibria of this cycle in both folded and unfolded forms of Rc and at two different pH values (2.5 and 7.0).

**Figure 6:**
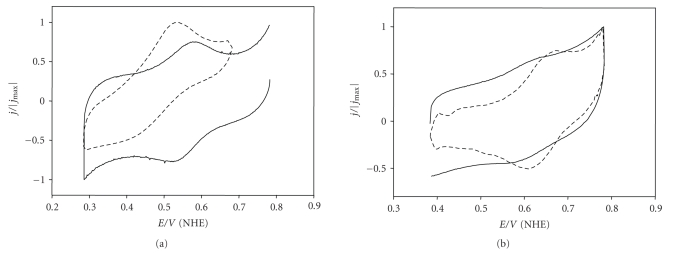
Cyclic voltammograms, expressed as normalized current density versus potential, of rusticyanin at (a) pH 7 and (b) pH 2.5. In both diagrams, solid and dashed lines correspond to the folded and unfolded forms of the protein, respectively. Conditions: Rc 0.1 M, phosphate buffer 0.1 M, and 298 K. For the unfolded forms, 8 M GdmCl was added.

**Figure 7:**
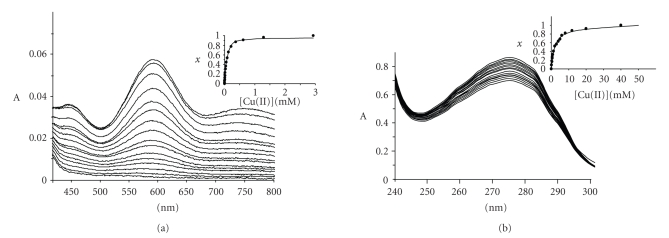
UV-visible titrations of apoRc as a function of the copper(II) concentration at pH 2.5 (298 K): (a) titration of the folded form (visible region); (b) titration corresponding to the unfolded form (UV region). The insets display the corresponding molar fractions (obtained from the absorbance at 592 and 270 nm for the folded and unfolded forms, resp.) as a function of the total copper(II) concentration.

Redox potentials were measured by means of cyclic voltametry. The cyclic voltammograms are displayed in Figure 6. At pH 2.5, the redox potential of the pair Cu(II)Rc/Cu(I)Rc (equilibrium 1) was 620 ± 10 mV, the same within the error previously reported [32]. The affinity constant of Cu(II) for apoRc (equilibrium 3) was measured by visible spectroscopy, following the formation of the blue charge transfer band characteristic of Rc at 592 nm (Figure 7(a)). A value of 3500 ± 150 M^−1^ was obtained. Variations of the Gibbs free energy, Δ*G* ^0^, for equilibria 1, and 3 of Scheme 1 result then to be −14.3 ± 1.1 and −4.8 ± 0.4 kcal/mol, respectively. By closing the cycle, a value of −15.6 ± 1.3 kcal/mol was obtained for Δ*G* ^0^_4_ , that is, an affinity constant of 3.11 × 10^11^ M^−1^ for apoRc for Cu(I) (the values of the equilibrium constants of reactions 3 and 4 are always referred to the formation of the corresponding holoforms, that is, as affinity constants, and, consequently, they are given in units of M^−1^).

**Figure 8:**
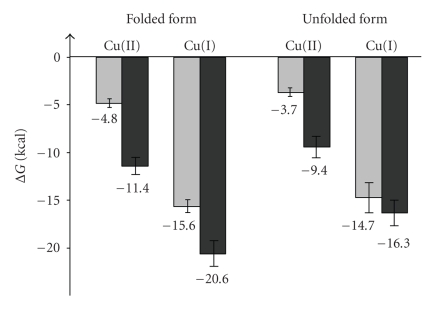
Gibbs free energies (in kcal/mol) corresponding to the formation of the complexes between copper (in both redox states) and the apoprotein (in the folded and unfolded forms), that is, corresponding to the equilibria (3) and (4) of Scheme 1. Light and dark grey bars correspond to data at pH 2.5 and pH 7.0, respectively. For the sake of clarity, the values of Δ*G* ^0^ associated to each process are explicitly indicated at the bottom of each bar.

Cycle of Scheme 1 can also be applied to the unfolded Rc at the same pH value (2.5). The redox potential of the pair Cu(II)/Cu(I)Rc for the unfolded protein has been measured, resulting to be 630 ± 10 mV (*G* ^0^_3_ = −14.7 kcal/mol). This value is slightly higher than that of the folded protein, indicating that, in these conditions, protein scaffold is less efficient in stabilizing copper(I) than the unfolded protein. The affinity constant of copper(II) for the unfolded form of Rc was determined by following the charge transfer band that appears at *ca.* 270 nm [12]. The obtained value was 500 ± 60 M^−1^ for the corresponding affinity constant (Figure 7(b)). Then the affinity constant of unfolded Rc for the reduced (copper(I)) species (equilibrium 4) can be deduced by closing the cycle. The resultant value was 6.55 × 10^10^ M^−1^ . Figure 8 shows the relative stabilities of the different holo species.

An analogous study was carried out at pH 7.0. The redox potential of the folded and unfolded forms of Rc are 550 (±5) and 450 (±15)mV, respectively. These values substantially decrease with regard to those obtained at pH 2.5 (see above). The redox potentials of folded Rc with the pH have been previously reported [32]. They are coincident with those here obtained. On the other hand, it is interesting to remark that redox potential of the unfolded rusticyanin at neutral pH is essentially the same to that obtained for unfolded azurin at the same experimental conditions (456 mV, [22]). This also corroborates that both azurin and rusticyanin possess the same coordination sphere in their unfolded forms.

Applying the same cycle (Scheme 1), the corresponding affinity constants can be obtained. In Figure 8, these values are displayed as well. As observed, the decrease in the redox potential at neutral pH is an immediate consequence of the high increment in the affinity of Rc for the copper(II) ion, from −4.8(± 0.4) to −11.4(±1.0) kcal/mol and from −3.7(± 0.5) to −9.4(+1.0) kcal/mol, for the folded and unfolded forms, respectively. This decrease is the result of a protonation/deprotonation residue close or, overall in the case of the unfolded protein, bound to the copper ion. From the copper ligands, only a histidine residue can undergo such a process in this pH range. Then the deprotonation of a histidine (necessarily HisC in the unfolded protein) is responsible for the decrease of the redox potential when the pH increases. In fact, a deprotonated histidine (with a lower positive charge) would present a higher affinity for the copper(II) ion, decreasing the redox potential.

It is interesting to remark that the folded species shows a manifestly higher affinity for the copper ion (in whatever oxidation state) than the unfolded form. This is more evident at pH 7.0 (see Figure 8). Rusticyanin is synthesized at the cytoplasm from *Af* at pH 6.5 and then secreted to the periplasm (pH *ca.* 3.0) [33]. Then, the present result can be relevant in vivo since free copper is found in an extremely low concentration inside the cell [2].

Finally, it is also noteworthy that the decrease in the redox potential is much more acute for the unfolded than for the folded form. This is a result of the different variation in the affinity of the reduced copper for the folded and unfolded Rc species (see Figure 8). While Cu(I) substantially increases its affinity for folded Rc when the pH rises, its affinity for unfolded Rc is not substantially altered. As a result, a drastic variation of the redox potential with the pH is observed. Then protein scaffold is acting as a “buffer” against a change in the charges of the media (i.e., aminoacids coordinated to the metal ion). When undergo protonation/deprotonation processes, the two histidines bound to the copper ion can interact with nearby residues of the active site, decreasing their net charge. Consequently, modifications in copper charge due to pH variations are also diminished.

## Figures and Tables

**Table 1:** Molecular weight and redox potentials of some representative BCPs [13].

Protein	Origin	Molecular Weight (kDa)	Redox Potential (mV)^a^
Halocyanin	*Natronobacterium pharaonis*	15.5	120
Stellacyanin	*Rhus vernicifera*	20.0	180
Auracyanin	*Chloroflexus aurantiacus*	12.8	240
Pseudoazurin	*Achromobacter cycloclastes*	13.4	260
Amycianin	*Thiobacillus versutus*	13.8	261
Azurin	*Alcaligenes denitrificans*	14.0	276
Umecyanin	*Armoracia laphatifolia*	14.6	283
Mavicyanin	*Cucurbita pepo medullosa*	18.0	285
Plantacyanin	*Cucumis sativus*	10.1	317
Plastocyanin	*Populus nigra*	10.5	370
Rusticyanin	*Acidithiobacillus ferrooxidans*	16.5	620

^a^Electrode potential values are referred to the normal hydrogen electrode (NHE) scale.
